# Reliability of Sensorimotor Control Tests in Individuals with Adolescent Idiopathic Scoliosis

**DOI:** 10.3390/muscles3040032

**Published:** 2024-11-15

**Authors:** Alexandros Kastrinis, Nikolaos Strimpakos, George A. Koumantakis, Dionysios Tzatzaliaris, Marianna Oikonomaki, Evangelos Theodosopoulos, Evangelia Skaftourou, Maria Tsekoura, Asimakis K. Kanellopoulos, Eleni Nomikou, Zacharias Dimitriadis

**Affiliations:** 1 Health Assessment and Quality of Life Research Laboratory, Department of Physiotherapy, Faculty of Health Sciences, University of Thessaly, 35132 Lamia, Greece; nikstrimp@uth.gr (N.S.); akanellopoulos@uth.gr (A.K.K.); zdimitriadis@uth.gr (Z.D.); 2 Research Laboratory of Advanced Physiotherapy, Department of Physiotherapy, University of Western Attica, 12243 Athens, Greece; gkoumantakis@uniwa.gr (G.A.K.); eleni_nomikou@yahoo.gr (E.N.); 3 Scoliosis Spine Laser Center, Moschato, 18345 Attica, Greece; Dtzatza@gmail.com (D.T.); mariannaoikonomaki@gmail.com (M.O.); 4 Athens Scoliosis-Spine Rehabilitation Clinic, Moschato, 18345 Attica, Greece; athensscoliosis@gmail.com (E.T.); evaskaf@hotmail.com (E.S.); 5 Physiotherapy Department, School of Health Rehabilitation Sciences, University of Patras, 26504 Rio, Greece; mariatsekoura@upatras.gr

**Keywords:** scoliosis, proprioception, joint position sense, repeatability, reproducibility, shoulder, knee, elbow, spine, balance, footplate, Fukuda, sensorimotor control

## Abstract

Background: The presence of sensorimotor control deficits in adolescent idiopathic scoliosis compared to typically developed adolescents is supported by the literature but lacks reliability studies for assessment in this population. This study aimed to assess the reliability of eight sensorimotor control tests, in terms of static and dynamic balance, joint position sense (JPS) tests of the extremities and the spine, and a functional upper extremity proprioceptive test in adolescent idiopathic scoliosis subjects. Methods: Sixty adolescent idiopathic scoliosis subjects were divided into four groups. Each group underwent two tests by the same examiner, repeated at 15 min intervals. Reliability was measured using the intraclass correlation coefficient (ICC), standard error of measurement (SEM), and smallest detectable difference (SDD). Results: The results showed high reliability for the upper extremity functional proprioception test, for the dynamic and static balance test, and for the spinal lateral flexion joint position sense test in both directions. On the other hand, the shoulder external rotation, knee extension, elbow flexion, and spinal flexion joint position sense tests demonstrated poor reliability in adolescent idiopathic scoliosis subjects. Conclusions: Therapists are recommended to use the functional upper extremity proprioception test, the Fukuda test, the static balance test using a force footplate, and the spinal lateral flexion joint position sense test for assessing treatment progress in this population.

## 1. Introduction

Adolescent idiopathic scoliosis (AIS) is a progressive condition that affects the spine, causing deformity in all three planes of movement [1]. The cause of idiopathic scoliosis remains unknown. However, numerous theories are believed to be implicated, such as related hormonal disorders and hereditary and genetic predisposition [2]. Central nervous system (CNS) disorders are also believed to be among the factors involved in the manifestation of AIS [3]. Individuals with AIS seem to have a disturbed perception of their spine deformities and consider their spine to be normally aligned [4]. They also seem to have a disturbed proprioceptive imprint of their body shape and position in space [5].

Proprioceptive organs like articular mechanoreceptors, skin mechanoreceptors, and muscle spindles are important for spatial joint position input and information on the speed and quality of movement [6]. Muscles play an important role in transmitting sensory feedback that contributes to motor control. In the musculotendinous junction, Golgi tendon organs (GTOs) monitor muscle tension and force production. Information from the muscle spindle and GTOs is transmitted to the central nervous system (CNS) [7]. With the integration of this information, the motor response is adjusted to ensure more coordinated and precise muscle activation [6].

Compared with non-scoliotic adolescents, research suggests that sensorimotor deficits exist in subjects with AIS, involving static and dynamic balance, lower extremity joint position sense, and cervical spine proprioception, when compared with their adolescent peers with typical spine growth [8,9]. However, research related to spinal proprioception is limited. Compared to healthy participants, Guyot et al. (2016) investigated AIS participants’ ability to accurately reposition their heads to a neutral orientation while being blindfolded using the cervicocephalic relocation test [10]. The authors reported the existence of proprioceptive impairments in scoliotic participants compared to non-scoliotic participants. Similar findings have also been reported regarding the upper and lower extremities, where scoliotic participants were less accurate in reproducing the targeted angle in the knee and elbow compared to healthy controls [11].

Wim Keessen et al. (1992) [12] used a spatial orientation test to examine the upper extremity proprioceptive accuracy in four different groups of patients (AIS patients with progressive curves and non-progressive spinal asymmetry, participants under nocturnal enuresis behavioral treatment, and healthy control). The results of this study suggested lower accuracy scores in both AIS and the spinal asymmetry group compared to the control groups. While sensorimotor control deficits in AIS patients have been clinically observed in terms of static and dynamic balance, kinesthesia, and joint position sense for the upper and lower extremities and the cervical spine, no reliability study has been found for tools that assess these deficits in scoliosis patients. Therefore, developing reliable assessment tools is not just beneficial but crucial for both clinical and academic purposes.

Proprioceptive and sensorimotor control strategies constitute essential elements in the therapeutic management of scoliosis, particularly through methodologies such as the Schroth technique and various physiotherapeutic scoliosis-specific exercises (PSSEs). These methodologies are designed to improve postural stability, enhance muscular strength, and rectify spinal irregularities by utilizing proprioceptive feedback and integrating sensorimotor functions [13].

Even though therapists use those strategies for therapeutic gains, reliable tools for this specific population permitting the assessment of treatments’ effectiveness in terms of sensorimotor control are missing. The development of such tools is necessary for clinical and academic purposes. Therefore, the purpose of this study was to determine, in AIS patients, the test–retest reliability of (1) an upper extremity functional proprioceptive test; (2) a joint position test for the shoulder external rotation, elbow flexion, and knee extension; (3) a joint position test for spinal flexion and lateral flexion; and (4) a dynamic and static balance test.

## 2. Materials and Methods

### 2.1. Sample

Sixty individuals diagnosed with AIS participated in this study. All volunteers were recruited from the patient pool of the Scoliosis Spine Laser Center and were assigned to four groups. Group A performed upper extremity proprioception tests while Group B performed dynamic and static balance tests. Group C focused on conducting spinal joint position sense tests in the sagittal and frontal planes. Finally, Group D performed knee and elbow joint position sense tests. Participants were included if they (a) were aged between 10 and 17 years, (b) had a Risser sign between 0 and 5, (c) had a main Cobb angle between 10° and 45°, and (d) were fluent in Greek. Volunteers were excluded if they suffered from (a) diseases that can affect motor control or (b) balance, (c) had cognitive problems or (e) mental illnesses, and (f) had vestibular pathologies not related to scoliosis. Before engaging in the study, all participants and their parents/guardians were presented with the study’s information sheet and had to sign a consent form. The study was conducted according to the guidelines of the Declaration of Helsinki and was approved by the Ethics Committee of the Physiotherapy Department, University of Thessaly, Lamia, Greece (234/5 April 2021).

### 2.2. Equipment and Materials

**Figure 1:**
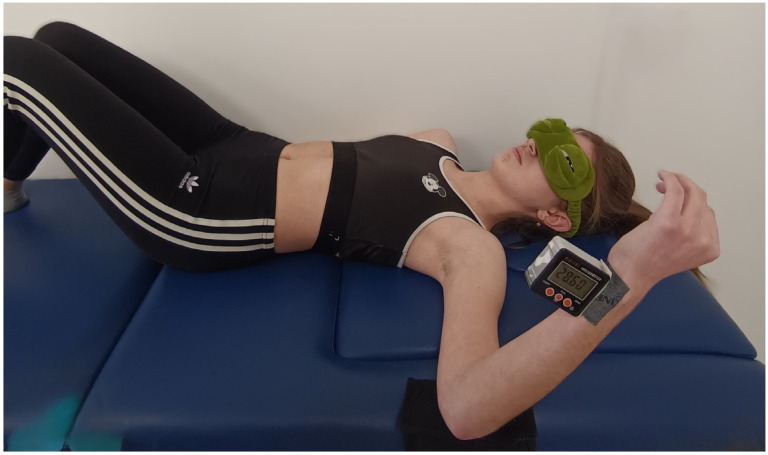
Shoulder external rotation joint position sense test. Target angle is set at 45°.

A digital inclinometer was used to assess the joint position sense (JPS) tests of the knee, elbow, and shoulder (Digital Level Box, eSync, Hong Kong, China). The inclinometer provides angle measurements in degrees (°) with an established accuracy of ±0.2 to 99 degrees (±0.1 degrees at 0 and 90 degrees) and with a resolution of 0.05 degrees. The device weighed 70 g and measured 6.6 × 6.5 × 3.4 cm. Double-sided adhesive tape (DS1925, HPX, Temse, Belgium) was used to stabilize the inclinometer on specific anatomical locations (Figure 1). Numerous studies used a digital inclinometer to assess joint positioning sense. Studies were conducted on the knee [14], shoulder, and elbow joints [15]. The reported interclass correlation coefficient (ICC) ranged from 0.967 to 0.981 for either the absolute or relative errors and the inter-tester reliability.

Reddy et al. (2020) [16] also investigated the intra- and inter-rater reliability of spinal JPS using a digital inclinometer. Sixty participants (30 with and 30 without low back pain). Two evaluators administered the assessments with sessions scheduled one day apart to appraise consistency and reliability. They reported robust reliability with the intra- and inter-rater ICC ranging from 0.75 to 0.92 and 0.75 to 0.93, respectively. Furthermore, participants with LBP demonstrated significantly greater proprioceptive errors than their healthy counterparts, thereby suggesting a compromised lumbar proprioceptive sense within the LBP group.

A blindfold, i.e., a one-size, adjustable face mask (Yunmoxiao, China), was used to negate vision during testing.

**Figure 2:**
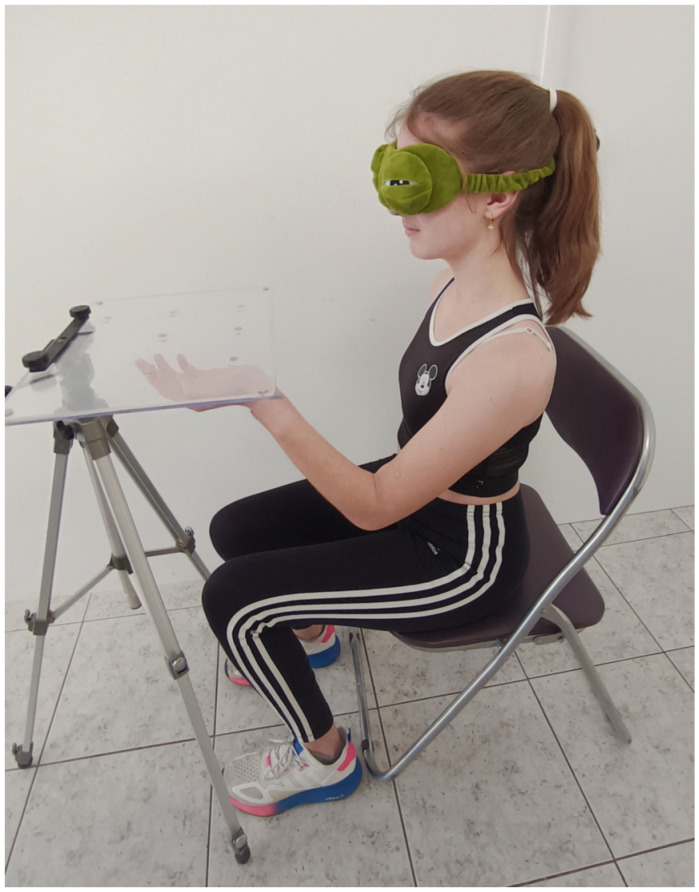
The participant places the left index finger in the hole under the transparent surface.

A panel-shaped device was used to assess the functional proprioception test for the upper limb (Figure 2). This device was initially used to assess proprioception in blind populations [17] and was later modified [12] to assess spatial orientation in individuals with AIS. The device (30 × 40 cm) consists of two transparent acrylic glass panels, bound together. The inferior panel was 5 mm thick, and the superior panel was 3 mm thick. Before binding, eight holes (8 mm) were made on the inferior panel to accommodate the participant’s index fingertip. The holes were made symmetrically from the center of the panel at a radius of 10 cm. When the two panels were bound together, the index finger that would be inserted from the inferior panel had no access to the surface of the superior panel. The device was then mounted on a tripod (Nedis TPOD2200GY, ’s-Hertogenbosch, The Netherlands) to adjust its height at the participant’s shoulder level. Holes 1, 4, and 6 were those closest and holes 3, 5, and 8 were those furthest from the participant’s torso. Holes 2 and 7 were mid-distance of the panel. A sewing thimble (Iris Sewing, China) covered the free arm index finger. A 2 mm hole was drilled on top of the thimble that would enable the assessor to mark with a pen the final resting position of the index finger placed on the upper panel.

Finally, a force footplate was used (model EPS+R, Loran Engineering, Bologna, Italy) to assess the mean sway velocity and center of gravity ellipse area in a standing position. The reliability of several center of pressure (COP) variables has been investigated [18] in two different standing conditions, with feet together and feet in a natural standing position. The study also investigated the optimal number of repetitions needed for a reliable outcome. Sixteen young adults with a mean age of 24.4 (±1.5) participated. The mean velocity and ellipse area had better reliability in a natural stance and with the three balance tests with a Cronbach’s of 0.9 and 0.78, respectively.

All distances were measured with SECA (Italy) measuring tape.

### 2.3. Procedure

After completing the demographic and informed consent forms, the volunteers were assigned to groups by picking a sealed envelope with their group designation.

This study focused on several sensorimotor control tests assessing balance and proprioception. For each experimental assessment, standardized protocols were implemented to minimize variability. Evaluations were performed with the eyes closed, after an initial trial conducted with the eyes open to facilitate participant comprehension of the required movements. Participants engaged in multiple trials of each assessment to ascertain trustworthy average measurements. For the joint position sense tests, the examiner passively guided the participant’s extremity or torso in achieving a designated position, which participants were subsequently instructed to replicate. The examiner quantified the discrepancy from the intended angle or position as a metric indicative of proprioceptive impairment or imbalance. A standard interval of 15 min between test repetitions was upheld to mitigate immediate learning effects, and a 20 min break was given between distinct tests to alleviate fatigue.

Group A participants performed the shoulder external rotation JPS test [19] and the upper extremity functional proprioception test. The shoulder external rotation JPS test was performed with the eyes closed and from a supine position with the knees bent. The examined shoulder was in an initial position of 90° abduction and 90° elbow flexion, with the forearm in a neutral position (Figure 1). The inclinometer was placed on the ulnar styloid process. The examiner passively guided the participant’s arm into 45° external rotation, maintaining the position for 5 s and asking the participant to memorize it. The participant was asked to repeat the movement afterward and reproduce the angle of 45°. The examiner measured the difference from the actual angle in each repetition in relation to the target angle. This procedure was repeated 5 times for each shoulder.

**Figure 3:**
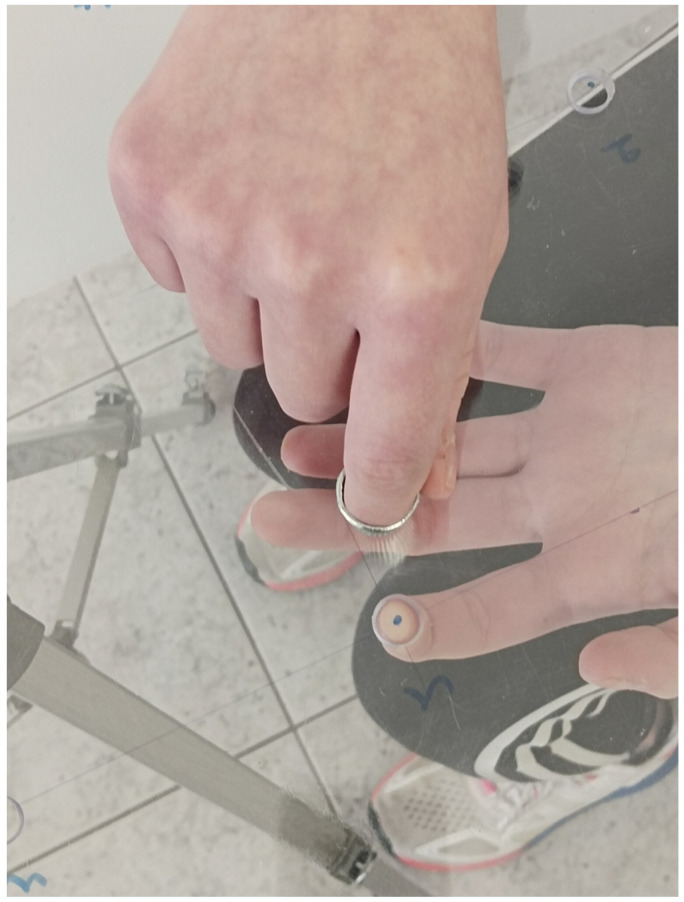
The participant tries to locate with the index finger of the “active” upper limb, from the top of the transparent surface, the exact position of the index finger of the hand that was previously placed in the hole under the transparent surface.

The second test followed after a 20 min break. The upper extremity functional proprioception test was performed seated with eyes closed. At shoulder height, in front of the participant, a transparent surface with 8 holes was placed. In random order, the index finger of one hand was placed in the hole from under the transparent surface. The participant was asked to locate, with his index finger on the other hand, from the top of the transparent surface, the exact position of the index of the hand that was previously placed in the hole [12]. As it was suggested in a previous study [11], a horizontal surface was chosen instead of a vertical one to avoid the possibility that similar muscle activation on both upper limbs may result in a better fingertip-matching end position. The examiner measured the distance between the participant’s index fingers each time (Figure 2 and Figure 3). This process was repeated 3 times for each of the 8 holes. The same measurements were made for both hands.

Group B participants performed the dynamic balance test first. To perform the Fukuda test, the initial standing position of the participant was marked. The participant was then asked to flex both shoulders to a 90° shoulder flexion position and maintain the position. With the eyes closed, the participant was asked to perform steps in place with a hip flexion angle of approximately 45°. After 50 steps, the distance travelled from the starting position was measured in centimeters, as well as the angle of rotation relative to the starting position [20]. The procedure was performed three times. Then, the participants would perform the static balance test on the force footplate. To measure the sway velocity and the center of gravity ellipse area, the participant stood shoeless on the force footplate with the heels separated by 10 cm. Participants were instructed to gaze at a fixed point in front of them at a distance of one meter [21].

Group C participants performed two spinal joint position sense tests, the trunk forward flexion and trunk lateral flexion tests. In the sagittal plane, forward flexion was assessed with two inclinometers placed over the C7 and L5 vertebrae. From a sitting position, the participant was instructed to maintain his pelvis in a neutral position and place his arms across his chest with the palms facing his shoulders. The patient was passively guided with a slow and steady pace to 20° of flexion with eyes open. The position was maintained for five seconds so that the patient could remember it, and then, the patient returned to the starting position [22]. The assessment was repeated five times. At each time, the deviation in the actual flexion angle compared to the one initially requested was noted.

The digital inclinometer was positioned over the C7 vertebra to perform the spinal lateral flexion joint position sense test. Using the same starting position and procedures, the participant was passively guided to 20° of spinal lateral flexion on the left and right.

Group D performed two joint position sense tests for the knee and elbow. The participants were seated with the popliteal area not touching the seat. Neoprene fabric pads were placed under the participant’s thigh to avoid proprioceptive input from skin mechanoreceptors. The initial positioning of the knee joint was at 90° flexion, with the inclinometer placed on the tibia. With their eyes closed, participants were instructed to maintain an upright sitting position. The examiner slowly (10°/s) passively moved the participant’s limb to 30° of flexion, where it remained for five seconds. The participant was asked to concentrate and memorize the position. Then, the leg was returned to its initial position. The procedure was repeated 5 times bilaterally. At each trial, the deviation between the actual angle that was achieved compared to the angle that was originally asked for was noted [23]. The procedure was repeated for the elbow. In a seated position with their eyes closed, the participant’s elbow was extended with the forearm in a neutral position. With the inclinometer positioned over the styloid process, the examiner slowly (10°/s) and passively moved the participant’s limb to the target angle of 45° of flexion, where it remained for five seconds. The participants were then instructed to concentrate and memorize the position. The procedure was repeated 5 times on both sides, and the deviation in the achieved angle compared to the original one was noted [11].

### 2.4. Data Analysis

Test–retest reliability for both tests was assessed based on the intraclass correlation coefficient (ICC), standard error of measurement (SEM), and smallest detectable difference (SDD). ICC values of 0–0.5, 0.5–0.75, 0.75–0.9, and 0.9–1 indicated poor, moderate, good, and excellent reliability, respectively [24]. Data were presented using means and standard deviations for continuous variables and percentages for categorical data. This approach afforded a comprehensive overview of central tendencies and variability, which are crucial for interpreting test–retest reliability outcomes. The standard error of measurement computation entailed extracting the square root of the average square within groups. The determination of the smallest detectable difference was achieved by utilizing the formula SEM multiplied by 1.96 and then by the square root of 2. The calculation of the SEM and SDD may seem somewhat subjective [25]; nevertheless, specific recommendations have been made for their clarification. Consequently, within the scope of this study, SEM values lower than 15% of the overall mean were considered acceptable [26]. Furthermore, SDD values under 30% were regarded as adequate, while those under 10% were classified as exceptional [25].

To further evaluate measurement error, we conducted an analysis of absolute and constant error types where applicable. Absolute errors were determined as the mean of the absolute differences between test and retest scores, thereby directly assessing measurement precision devoid of directional bias. Constant errors, indicative of the average directional discrepancy between test and retest scores, highlighted the existence of systematic biases within the measurements [27,28]. IBM SPSS for Windows, version 25.0 (IBM Corp., Armonk, NY, USA), was used for all statistical analyses.

## 3. Results

The sample consisted of sixty participants with a mean age of 14.02 (SD 1.6) who were equally divided into four groups. All participants were undergoing bracing and Schroth treatment. Between testing and retesting, no treatment was applied. The average height and weight were, respectively, 162.2 (SD 1.6) and 49.4 (SD 7.5). The participants’ mean Cobb angle was 25.3° (SD 7.1°) (Table 1).

Group A performed two tests. The test–retest reliability for the shoulder external rotation position sense test (Table 2) was found to be poor for both the absolute test error for the right- (ICC (95% CI) = 0.18 (0–0.71), SEM = 4.16, SDD = 11.54) and left-side shoulder (ICC (95% CI) = 0.37 (0–0.77), SEM = 3.59, SDD = 9.96) and for the continuous error for the right- (ICC (95% CI = 0.57 (0–0.86), SEM = 5.52, SDD = 15.31) and left-side shoulder (ICC (95% CI) = 0.59 (0–0.85), SEM = 5.19, SDD = 14.38).

The test–retest reliability of the upper extremity functional proprioception test (Table 2) was found to be good to excellent for both the total test score (ICC (95% CI) = 0.90 (0.71–0.96), SEM = 0.47, SDD = 1.3) and individually for the left (ICC (95% CI) = 0.86 (0.40–0.91), SEM = 0.47, SDD = 1.3) and right upper extremity (ICC (95% CI) = 0.87 (0.63–0.95), SEM = 0.67, SDD = 1.87). The statistical means for each individual side (left and right) as well as the joint averages (R + L) were derived as the overall means of the separate measurements (H1mean through H8mean). The conclusions drawn are based upon the total means.

Group B participants performed two tests related to dynamic and static balance. The Fukuda test (Table 3) was found to have good test–retest reliability for the component that measured the distance from the starting point. According to the results, the means of the second and third trial of the absolute error measurement appeared to have better reliability (ICC (95% CI) = 0.85 (0.56–0.85), SEM = 15.03, SDD = 41.64).

Regarding the test for the static balance (Table 3), the means of the first two trials showed good reliability (ICC (95% CI) = 0.74 (0.23–0.91), SEM = 6.37, SDD = 17.66) for the sway velocity and (ICC (95% CI) = 0.74 (0.27–0.91), SEM = 142.46, SDD = 394.43) for the COG ellipse area.

Group C performed two tests related to the spinal position sense in the sagittal and the frontal planes. The spinal flexion joint position sense test (Table 4) has shown poor test–retest reliability, both for the constant error (ICC (95% CI) = 0.56 (0–0.85), SEM = 3.62, SDD= 10.04) and for the absolute error (ICC (95% CI) = 0.49 (0–0.83), SEM = 2.70, SDD = 7.49).

The spinal lateral flexion joint position sense test (Table 4) showed good to excellent test–retest reliability for the constant and absolute error on both sides (left side constant error (ICC (95% CI) = 0.83 (0.5–0.94), SEM = 1.50, SDD = 4.16), left side absolute error (ICC (95% CI) = 0.84 (0.53–0.94), SEM = 0.93, SDD = 2.59), right side constant error (ICC (95% CI) = 0.95 (0.85–0.98), SEM = 1.53, SDD = 4.24), right side absolute error (ICC (95% CI) = 0.94 (0.83–0.98), SEM = 1.45, SDD = 4.01)). Nevertheless, we calculated that 10 trials would be required for this test in order to achieve an SEM value equal to the 20% of the grand mean value.

Participants in Group D were tested for the joint position sense for the knee and elbow. The left-side knee joint position sense test (Table 5) had good test–retest reliability for the constant error (ICC (95% CI) = 0.76 (0.28–0.92), SEM = 4.38, SDD = 12.15), but the SEM and SDD values were not acceptable. For the absolute error on the same side, the test–retest reliability was poor (ICC (95% CI) = 0.56 (0–0.74), SEM = 3.58, SDD = 9.91). On the right side, both for the constant (ICC (95% CI) = 0.33 (0–0.78), SEM = 5.07, SDD = 14.05) and the absolute error (ICC (95% CI) = 0.32 (0–0.77), SEM = 3.70, SDD = 10.25), the test–retest reliability was poor. 

Regarding the elbow joint position sense test (Table 5), the left side has shown poor test–retest reliability for the constant error (ICC (95% CI) = 0.40 (0–0.8), SEM = 6.11, SDD = 16.94). For the absolute error on the same side, the test–retest reliability was poor (ICC (95% CI) = 0.17 (0–0.70), SEM = 4.12, SDD = 11.43). On the right side, both for the constant (ICC (95% CI) = 0.43 (0–0.81), SEM = 5.8, SDD = 16.06) and the absolute error (ICC (95% CI) = 0.18 (0–0.72), SEM = 4.5, SDD = 12.47), the test–retest reliability was poor.

## 4. Discussion

Eight sensorimotor control tests have been studied for their test reliability in scoliotic population. Four tests, namely the Fukuda test, the static balance test, the spinal lateral flexion JPS test, and the upper extremity functional proprioception test demonstrated high test–retest reliability. The discrepancy in reliability between these eight tests may have significant implications for clinical and research purposes. The high reliability of those four tests makes them valuable tools for assessing sensorimotor control and monitoring changes over time in various settings. In contrast, the poor reliability of the shoulder external rotation JPS test, knee extension JPS test, elbow flexion JPS test, and spinal flexion JPS test raises concerns about their suitability for assessing proprioception in terms of joint position sense accurately.

This study’s purpose was to evaluate the test-rest reliability of eight sensorimotor control tests for the upper and lower extremities, the spine, and the balance of individuals with AIS. Those tests were designed to assess sensorimotor control in terms of static and dynamic balance, proprioception, kinesthesia, and joint position sense. To our knowledge, this study is the first to examine test–retest reliability in this population.

Assessing sensorimotor control is complex, and no single tool serves as the “gold standard” for this kind of assessment. In the literature, several tools are used, including motion analysis, goniometers, isokinetic dynamometers, inclinometers, or other tools [29]. The reasoning for the development of the tools in this study was to enable therapists to monitor treatment progress in idiopathic scoliosis patients in terms of somatosensory deficits in a clinical setting with easy-to-use equipment.

In the domain of sensorimotor control, proprioception is a very complex neurophysiological process. Proprioception, in conjunction with other senses, plays a critical role both in feedback and feed-forward mechanisms. Due to this complexity, measuring proprioception is very challenging [30]. In clinical settings, proprioceptive tools have been poorly assessed for their reliability. Prior studies have mentioned between two and six trials when JPS is being assessed [31]. The authors’ main concern regarding multiple trials is muscle fatigue, which might lead to a decrease in proprioception and a learning effect improving the measurements [32,33]. A comprehensive systematic review [34] indicates that averaging the outcomes of three to five repetitions conducted on a stable surface is imperative to achieve satisfactory reliability concerning center of pressure assessments in static balance tasks. Barisic et al. (2023) examined this phenomenon in adolescents and reported that a minimum of three trials may be necessary for male participants to achieve stabilization attributable to familiarization. This learning process was less for females [35].

The upper extremity functional proprioception test demonstrated excellent reliability. Moreover, the SEM and SDD suggested a small margin of error and the ability to detect minor changes in proprioception. This was also noted for each upper extremity, with the right side displaying slightly more variability based on higher SEM and SDD values. According to one study [12], the normal population is more accurate while performing a similar test. The researchers report a significant inaccuracy for right-handed participants with scoliosis or spinal asymmetries. In our study, even though the right-hand reliability was strong, higher variability was detected.

On the other hand, the shoulder external rotation joint position sense test showed poor reliability. Having the fatigue factor in mind [32,33], analyses have been conducted for trials 1–3 as well as for the total set of five trials. The ICC values for absolute and continuous errors were notably low, indicating inconsistent reproducibility. Values for absolute and constant error for the right and left shoulder do not meet the criteria for good reliability [27,28], suggesting limitations in detecting proprioceptive changes. In the literature, external rotation JPS is evaluated from several positions [31,36]. In this study, the supine position was chosen for increased stability. The lower reliability reported in our study might be attributed to the altered joint alignment seen in scoliosis patients as individuals with a thoracic curve tend to have a protracted shoulder position on the convex side of the curve and a retracted shoulder position on the concave side of the curve [37]. This leads to altered shoulder kinematics. It is suggested that as an attempt of the body to adapt to the spinal deformity caused by scoliosis, the length–tension curves of the muscles surrounding the shoulder area are altered, which could lead to a different muscle activation pattern [38]. Group D also performed two joint position sense tests for the upper and lower limbs (knee and elbow). Like the JPS test for shoulder external rotation, the ICC values for absolute and continuous errors bilaterally were notably low, indicating inconsistent reproducibility. These results also suggest limitations in detecting proprioceptive changes. This might be due to the choice for testing in the mid-range for all tested joints. End-range positions might give better results of position sense [36,39]. A study that assessed JPS in the knee [40] suggests that proprioceptive acuity was different in various target positions. Two more studies reported better movement sense in the shoulder area, especially at the end of the range of motion [6,41].

According to a recent literature review [29], the inter- and intra-rater reliability of the shoulder external rotation JPS test show promising results. However, these studies did not involve individuals with AIS. The elbow test–retest had moderate to good reliability in healthy adults and especially in the 60° target angle [42], in contrast with our study. Regarding the reproducibility of the knee JPS test, a recent study [43] in a healthy population reported low reliability at the 450 target angle. In terms of muscle receptors, the consistency of the feedback provided during the test and retest trials would be helpful. Input regarding muscle length and tension from the muscle spindles and GTOs to the CNS should be accurate. This stable sensory input is crucial for the CNS to interpret and generate motor commands with precision. The outcome for the reliability of the upper extremity functional proprioception test might be due to the fact that a broader limb movement is tested, allowing proprioceptive feedback from multiple joints and skin receptors [44]. This could have allowed compensation for possible deficits, thus permitting better performance consistency. The JPS test of the shoulder external rotation, the knee extension, and the elbow flexion isolate a specific movement, and a more precise neuromuscular control is required in addition to a level of proprioceptive input that individuals with scoliosis seem to lack. Group C also performed JPS reliability tests for spinal flexion and the spinal lateral flexion. Dimitriadis et al. (2022) [45] assessed the reliability of spinal flexion with a double inclinometer method. The participants of the study were adults with a history of low back pain. The test–retest reliability testing was performed in the standing position, and according to the results, it was poor (ICC (95% CI) = 0.15 (0–0.67), SEM 3.98, SDD 11.02). In our study, the sitting position was chosen to ensure better pelvic control while the spine is still loaded, since the nature of the three-dimensional deformity can lead to core muscle strength imbalances and postural deviations [46]. Testing the thoracic and lumbar segments of the spine in sagittal and frontal planes was chosen because of the clinical implication in AIS and its ability to affect all spinal segments. In this study, the spinal flexion JPS test has shown poor reliability. Even though participants were instructed to maintain an upright starting position and a neutral pelvis, it was noticed by the assessor that most of them failed to do so. On the other hand, the lateral flexion JPS test has shown good to excellent test–retest reliability for the constant and absolute error on both sides (Table 4). From our results, ten trials would be needed to achieve an SEM value equal to 20% of the grand mean value. This calculation was made using the Spearman–Brown formula [47] to avoid a big margin of error.

Group B has performed two balance tests to assess the repeatability of a dynamic balance test and a static balance test, and the Fukuda test was found to have good reliability in the distance from the starting point component. In a healthy population study [48], the Fukuda test was found to have adequate reliability (ICC = 0.66) for the final angle in relation to the starting position and (ICC = 0.69) for the distance from the starting position in the 50-step protocol. Regarding the test–retest reliability of the static balance test in healthy students aged 6 to 14 years, moderate reliability (ICC = 0.57 and 0.61) for the COG ellipse area with the eyes open and closed, respectively, and very good reliability (ICC = 0.75 and 0.76) for the sway velocity with the eyes open and closed, respectively, were reported [49]. The findings in our study also showed good reliability.

The results in this study reveal significant variations in consistency across multiple anatomical regions. With respect to the demographic attributes among the four cohorts, the parameters of age, height, and weight exhibit a considerable degree of similarity across the groups. The Cobb angles recorded for the participants are also comparable, implying that variances in the severity of spinal deformities do not constitute a primary factor for any discrepancies in the reliability of the tests. It is improbable that any variations in test outcomes are significantly affected by these demographic distinctions.

The balance assessments for Group B and the upper extremity evaluation for Group A pertain to functional proprioception, an all-encompassing dynamic component. A comparative analysis may be feasible from this perspective, focusing on the consistency of the tests and the insights provided by the SEM and SDD values regarding the reliability of each assessment. The upper extremity functional proprioception evaluation for Group A demonstrates the highest level of reliability (ICC = 0.90). This finding indicates that participants’ capacity to consistently perceive upper limb positions is relatively stable. The Fukuda test in Group B, which quantifies balance by evaluating the extent of deviation while marching in place, also exhibits robust reliability (ICC = 0.85). Conversely, the footplate assessment in Group B, which measures sway velocity during a state of quiet standing, presented somewhat diminished reliability (ICC = 0.74). The SEM for Group A of 0.47 indicates minimal measurement error for upper extremity proprioception. In contrast, the SEM associated with the balance assessments in Group B is greater (15.03 for Fukuda and 6.37 for footplate), suggesting larger variability in participants’ balance performance across trials. The SDD values elucidate the magnitude of change required in scores between test sessions to be deemed beyond measurement error. Group A has an SDD of 1.3, indicating that alterations smaller than this threshold are likely attributable to random variability. For Group B, the SDDs are considerably higher (41.64 for Fukuda and 17.66 for sway velocity), signifying that more substantial changes would be necessary to assert with confidence that there is a genuine difference in performance over time. In a clinical context, it is advisable for practitioners to use both balance assessments to achieve a more comprehensive understanding of the patients’ proprioceptive capabilities concerning balance.

In the present investigation, the reliability of joint position sense (JPS) assessments across various joints (namely shoulder, spinal, knee, and elbow) and the effect of these findings for clinical applications or prospective research endeavors are analyzed. The JPS associated with the spinal region (Group C) exhibits the highest degree of reliability (ICC = 0.83–0.95) specifically for lateral flexion movements. This observation may suggest that the spinal JPS is comparatively easier for participants to execute with consistency, potentially reflecting an enhanced level of proprioceptive control within this anatomical area. Conversely, the spinal JPS assessments conducted in the sagittal plane demonstrated a lack of consistency. The assessments related to the shoulder (Group A) and the elbow and knee (Group D) display diminished ICC values, which implies a greater degree of variability in joint position sense for these particular joints. Standard error of measurement (SEM) and smallest detectable change (SDD): The SEM for spinal lateral flexion JPS is notably low (1.50–1.53), indicating that the measurements possess a high degree of precision. Furthermore, the SDD is also relatively small (4.01–4.24), signifying that even minimal alterations in spinal proprioception may hold significant implications. Conversely, for the shoulder, elbow, and knee, the elevated SEM and SDD values denote an increased measurement error and a diminished level of precision.

In future research, emphasis should be placed on spinal lateral flexion JPS, upper extremity proprioception, and balance assessments due to their demonstrated reliability. Future investigations focusing on spinal flexion in the sagittal plane, in addition to elbow, knee, and shoulder JPS assessments, should focus on the number of repetitions, the sensitivity of the equipment utilized, and modifications to the protocol to enhance reliability.

The assessments indicating diminished reliability (e.g., shoulder external rotation joint position sense (JPS), knee and elbow JPS, spinal flexion JPS) warrant additional investigation. Numerous elements may have influenced these results: Proprioception is facilitated by an integrative network of joint, muscle, and cutaneous receptors. The complexity of these systems can result in inconsistencies in JPS reliability. For example, it has been suggested that muscle receptors significantly contribute to proprioception, occasionally surpassing the influence of joint receptors, thereby complicating the sensory feedback mechanism [50]. Clinical evaluations of joint position sense frequently depend on subjective approaches, such as limb matching responses, which may inadequately quantify proprioceptive impairments. Variations in testing conditions, including active versus passive movements, weight-bearing versus non-weight-bearing stances, and the effects of adjacent joint positioning, can substantially influence JPS assessment results [51]. Variations in body posture can also modify the perception of joint position sense. For instance, modifications in head and neck alignment have been documented to affect elbow joint position sense, implying that analogous positional variations could alter proprioception in individuals with scoliosis [52]. Joint laxity has been correlated with heightened trunk rotation in scoliosis patients. This correlation indicates that ligamentous laxity may contribute to alterations in spinal contour and potentially influence proprioceptive feedback [53]. Future studies might contemplate modifications in testing protocols, enhancement of equipment sensitivity, or the exploration of alternative measurement methodologies. In this direction, an enhanced apparatus or motion-tracking technologies exhibiting superior sensitivity to accurately capture nuanced joint movements could be used. Optimizing testing methodologies to incorporate an increased number of practice trials for participants prior to formal assessment may contribute to the reduction in variability and the enhancement of reliability.

This study has some limitations. The sample size in this study was not determined through statistical calculations, which may give the impression of being relatively small. Τhis may restrict the statistical power and generalizability of our results. In any case, the number of samples was limited by practical and logistical challenges. Even though studies with small sample sizes do exist [54], future studies should endeavor to recruit a larger and more heterogeneous sample to augment the statistical power and generalizability of the outcomes [14]. This may necessitate multi-site collaborations or prolonged recruitment durations to ensure a greater number of volunteers are integrated. Executing a power analysis to ascertain the optimal sample size for identifying significant effects would also be advantageous. This would assist in directing future studies and guaranteeing that sufficient statistical power is attained [14]. Regarding equipment sensitivity, future studies should include the standardization of the measurement protocol and the execution of inter-rater reliability assessments to ensure consistency across measurements, which is imperative [55]. More sensitive measurement instruments may facilitate the utilization of reduced sample sizes while still attaining substantial reliability assessments [56]. Furthermore, more extensive training for data collectors could diminish procedural variability. An additional limitation pertains to the retest session for both tools of every group, being scheduled on the same day with a minimal interval between the initial test and the retest. This choice could potentially contribute to unsatisfactory test–retest reliability due to possible fatigue effects. However, the rationale behind this interval selection was primarily aimed at minimizing drop-out rates and enhancing control over patients’ activities between the testing sessions. Nevertheless, in some circumstances, same-day test–retest reliability is acceptable [57]. 

## 5. Conclusions

Four sensorimotor control tests (the upper extremity functional proprioception test, The Fukuda test, the static balance test, and the lateral flexion JPS test] demonstrate a high level of reliability in individuals with adolescent idiopathic scoliosis. The upper extremity functional proprioception test should be performed three times for each hole, while for the Fukuda test, the means of the second and third trials should be considered.

For the static balance test, two trials are adequate. For the lateral flexion JPS test, according to calculations, the mean of ten trials is necessary for a reliable measurement. Clinicians seeking to assess the progress of therapy with regard to sensorimotor control could employ these particular evaluation tools. As far as the authors are aware, these tools represent the first dependable instruments for appraising sensorimotor control in those diagnosed with adolescent idiopathic scoliosis.

In contrast, the assessment of the shoulder external rotation JPS test, knee extension JPS test, elbow flexion JPS test, and spinal flexion JPS test in individuals affected by adolescent idiopathic scoliosis exhibits limited reliability. It is advised against the utilization of these evaluation methods by therapists for monitoring proprioception in terms of joint position sense throughout the treatment process.

Identifying dependable sensorimotor control assessments offers clinicians the opportunity for the appraisal of therapeutic advancements in patients with adolescent idiopathic scoliosis (AIS) with robust instruments. The utilization of reliable assessments mitigates the probability of divergent results. This will help the decision-making process, regarding modifications to treatment protocols. This study outlines critical reliability data for sensorimotor assessments in AIS, addressing a notable gap in scoliosis research.

Further investigations ought to pursue the refinement and validation of supplementary reliable assessments concerning proprioceptive control in individuals diagnosed with adolescent idiopathic scoliosis (AIS). An examination of more specific modifications of joint position sense (JPS) assessments or the incorporation of innovative technologies, such as motion capture systems or wearable sensor devices, may significantly improve the precision of measurements. Longitudinal research scrutinizing the correlation between these reliable assessments and clinical outcomes throughout the scoliosis treatment continuum would yield valuable insights for the customization of patient-centered therapeutic strategies.

## Figures and Tables

**Table 1:** Participant demographics and clinical characteristics (mean (SD)).

Parameter	Population	Group A	Group B	Group C	Group D
Gender (Female) n (%)	48 (60)	48 (60)	48 (60)	48 (60)	48 (60)
Age (Years)	14.02 (1.6)	14.3 (1.5)	13.8 (1.6)	13.86 (1.74)	14.13 (1.84)
Height (cm)	162.2 (9.7)	164.06 (11.43)	157.8 (9.4)	163.06 (7.14)	164.2 (10.95)
Weight (kg)	49.4 (7.5)	49.06 (7.57)	48.1 (6.7)	48.7 (8.67)	51.8 (7.32)
Cobb angle (Primary Curve)	25.3° (7.1)	23.8 (5.56)	25.6 (8.65)	25.6 (8.42)	26.2 (6.0)
**Primary Curve**					
Thoracic	26	5	9	7	5
Lumbar	30	8	6	7	8
Thoracolumbar	4	2	0	0	2

**Table 2:** Group A. Upper extremity proprioception tests.

Test–Retest Reliability for the Shoulder External Rotation Position Sense Test							
Side	Type of Error	Trials	GM	ICC	95%CI	SEM	SDD
R	Absolute	Mean1-3	6.69	0.14	0–0.71	4.56	12.64
		Mean1-5	6.5	0.18	0–0.71	4.16	11.54
	Constant	Mean1-3	−3.53	0.55	0–0.85	5.92	16.4
		Mean1-5	−2.87	0.57	0–0.86	5.52	15.31
L	Absolute	Mean1-3	5.59	0.23	0–0.72	3.73	10.33
		Mean1-5	6.07	0.37	0–0.77	3.59	9.96
	Constant	Mean1-3	−0.05	0.57	0–0.84	4.91	13.6
		Mean1-5	−0.09	0.59	0–0.85	5.19	14.38
**Test–Retest Reliability of the Upper Extremity Functional Proprioception Test**							
**Side**			**GM**	**ICC**	**95%CI**	**SEM**	**SDD**
L	Total mean		1.91	0.86	0.40–0.91	0.47	1.3
R	Total mean		2.8	0.87	0.63–0.95	0.67	1.87
Both (R + L)	Total mean		2.39	0.90	0.71–0.96	0.47	1.3

GM: grand mean; ICC: intraclass correlation coefficient; 95%CI: 95% confidence interval; SEM: standard error of measurement; SDD: smallest detectable difference.

**Table 3:** Group B. Dynamic and static balance tests.

Test–Retest Reliability for the Fukuda Test								
Outcome Measure	Type of Error	Trials		Grand Mean	ICC	95%CI	SEM	SDD
Angle from the baseline (^o^)	Absolute	Mean1-2		13.98	0.14	0–0.56	11.57	32.05
		Mean2-3		14.08	0.52	0–0.84	8.86	24.55
		MeanAll		14.73	0.52	0–0.84	9.41	26.07
	Constant	Mean1-2		6.98	0.44	0–0.81	19.59	54.28
		Mean2-3		6.33	0.44	0–0.81	20.30	56.23
		MeanAll		5.86	0.48	0–0.83	19.91	55.16
Distance from the baseline (cm)	Absolute	Mean1-2		52.8	0.85	0.29–0.95	12.2	33.8
		Mean2-3		47.53	0.85	0.56–0.95	15.03	41.64
		MeanAll		50.66	0.88	0.44–0.96	11.67	32.33
	Constant	Mean1-2		52.06	0.84	0.23–0.95	13.03	36.01
		Mean2-3		47	0.84	0.52–0.94	15.61	43.26
		MeanAll		50	0.87	0.37–0.96	12.17	33.71
**Test–Retest Reliability for the Footplate Analysis**								
**Outcome Measure**	**Trials**		**Grand Mean**		**ICC**	**95%CI**	**SEM**	**SDD**
Mean of sway velocity (mm/s)	1-2		17.61		0.74	0.23–0.91	6.37	17.66
	2-3		18.69		0.44	0–0.81	8.34	23.12
	All		17.68		0.68	0.01–0.89	5.71	15.8
COG Ellipse Area (mm^2^)	1-2		267.28		0.74	0.27–0.91	142.46	394.63
	2-3		290.02		0.63	0–0.87	177.13	490.65
	All		264.57		0.68	0.06–0.89	139.2	385.09

GM: grand mean; ICC: intraclass correlation coefficient; 95%CI: 95% confidence interval; SEM: standard error of measurement; SDD: smallest detectable difference. COG: center of gravity.

**Table 4:** Group C. Spinal joint position sense tests in sagittal and frontal planes.

Test–Retest Reliability for the Spinal Flexion Joint Position Sense Test							
Type of Error		Trials	GM	ICC	95%CI	SEM	SDD
Constant		Mean1-3	−3.98	0.58	0–0.86	3.34	9.26
		Mean1-5	−3.53	0.56	0–0.85	3.62	10.04
Absolute		Mean1-3	4.89	0.53	0–0.84	2.73	7.56
		Mean1-5	4.99	0.49	0–0.83	2.70	7.49
**Test–Retest Reliability for the Spinal Lateral Flexion Joint Position Sense Test**							
**Side**	**Type of Error**	**Trials**	**GM**	**ICC**	**95%CI**	**SEM**	**SDD**
L	Constant	Mean1-3	0.95	0.77	0.29–0.85	1.57	4.37
		Mean1-5	1.58	0.83	0.50–0.94	1.50	4.16
	Absolute	Mean1-3	2.48	0.71	0.12–0.90	1.01	2.81
		Mean1-5	2.99	0.84	0.53–0.94	0.93	2.59
R	Constant	Mean1-3	2.54	0.90	0.72–0.96	1.99	5.53
		Mean1-5	3.18	0.95	0.85–0.98	1.53	4.24
	Absolute	Mean1-3	3.52	0.90	0.69–0.96	1.78	4.93
		Mean1-5	3.95	0.94	0.83–0.98	1.45	4.01

GM: grand mean; ICC: intraclass correlation coefficient; 95%CI: 95% confidence interval; SEM: standard error of measurement; SDD: smallest detectable difference.

**Table 5:** Group D. Knee and elbow joint position sense tests.

Test–Retest Reliability for the Knee Joint Position Sense Test							
Side	Type of Error	Trials	GM	ICC	95%CI	SEM	SDD
L	Constant	Mean1-3	3.84	0.80	0.26–0.87	3.69	10.23
		Mean1-5	4.84	0.76	0.28–0.92	4.38	12.15
	Absolute	Mean1-3	6.12	0.70	0.17–0.89	2.59	7.19
		Mean1-5	7.22	0.56	0–0.74	3.58	9.91
R	Constant	Mean1-3	3.51	0.21	0–0.74	5.02	13.91
		Mean1-5	4.26	0.33	0–0.78	5.07	14.05
	Absolute	Mean1-3	5.25	0.46	0–0.82	3.12	8.65
		Mean1-5	5.83	0.32	0–0.77	3.70	10.25
**Test–Retest Reliability for the Elbow Joint Position Sense Test**							
**Side**	**Type of Error**	**Trials**	**GM**	**ICC**	**95%CI**	**SEM**	**SDD**
L	Constant	Mean1-3	2.94	0.34	0–0.76	6.17	17.09
		Mean1-5	2.86	0.40	0–0.80	6.11	16.94
	Absolute	Mean1-3	7.22	0.17	0–0.70	4.12	11.43
		Mean1-5	7.04	0.11	0–0.68	4.06	11.25
R	Constant	Mean1-3	5.2	0.51	0–0.83	5.66	15.68
		Mean1-5	5.76	0.43	0–0.81	5.8	16.06
	Absolute	Mean1-3	7.32	0.21	0–0.73	4.76	13.19
		Mean1-5	7.33	0.18	0–0.72	4.5	12.47

GM: grand mean; ICC: intraclass correlation coefficient; 95%CI: 95% confidence interval; SEM: standard error of measurement; SDD: smallest detectable difference.

## Data Availability

Data are unavailable due to privacy and ethical restrictions.
